# Monitoring and Volatile Profiling of Fruit Crops as Host Plants of the Polyphagous Brown Marmorated Stink Bug *Halyomorpha halys* (Stål, 1855)

**DOI:** 10.3390/insects17020186

**Published:** 2026-02-10

**Authors:** Alicia Koßmann, Bruna Czarnobai de Jorge, Asya Demir, Astrid Eben, Jürgen Gross

**Affiliations:** 1 Institute for Plant Protection in Fruit Crops and Viticulture, Julius Kühn Institute, Federal Research Centre for Cultivated Plants, Schwabenheimer Str. 101, 69221 Dossenheim, Germany; bruna.czarnobai@julius-kuehn.de (B.C.d.J.); astrid.eben@julius-kuehn.de (A.E.); 2 Plant Chemical Ecology, Technical University of Darmstadt, Schnittspahnstr. 4, 64287 Darmstadt, Germany; 3 Department of Crop Protection, Hochschule Geisenheim University, Von-Lade-Str. 1, 65366 Geisenheim, Germany

**Keywords:** attractants, hemiptera, pentatomid, headspace-sampling, volatiles, monitoring, GC-MS, olfactometer, electroantennography

## Abstract

Volatile organic substances (VOCs) emitted by plants play an important role in the recognition and selection of host plants by insects. For polyphagous insects with a broad host range, like the brown marmorated stink bug *Halyomorpha halys*, not much is known about the plant volatiles that influence host choice. In order to determine which odour stimuli could influence host selection, monitoring was carried out using pheromone traps in orchards with various host plants. The headspace of the phenological stages of plants on which *H. halys* occurred in large numbers was sampled and analysed with gas chromatography coupled with mass spectrometry (GC-MS). The volatile profiles of the different host plants varied significantly. Some compounds occurred in high relative proportions across all taxa. Those compounds were tested by *H. halys* using electroantennography. *H. halys*’ antennae responded significantly to all of the selected compounds. In a Y-tube olfactometer, *H. halys* showed a significant attraction to 1 µg hexanal, 100 µg (*E*)-4,8-dimethyl-1,3,7-nonatriene (DMNT), and a volatile mixture. Due to the limited sustainable strategies for plant protection against this polyphagous insect, adding attractive plant volatiles to lures could improve the effectiveness of alternative volatile-based plant protection strategies, such as traps or capsules, or promote their development.

## Simple Summary

The invasive, polyphagous brown marmorated stink bug (*Halyomorpha halys*) can cause serious damage to various crops. Understanding how it selects its host plants is important for developing new pest management strategies. This study explored the role of plant volatile organic compounds in attracting the stink bug to certain plants. For this, monitoring of various fruit crop was conducted, and the volatiles of different hosts were collected and analyzed using advanced techniques to identify the chemicals involved. Common compounds across several plant species were identified and the stink bug responded strongly to them. In behavioural tests, the bug was attracted to single compounds and a mixture of specific volatiles. Plant protection strategies against this pest are limited. Using attractive scents could improve pest control methods and offer a more sustainable alternative to traditional chemical pesticides. This research aims to create more effective and environmentally friendly strategies for managing *H. halys* in horticulture and fruticulture.

## 1. Introduction

Globalisation and climate change have led to the invasion of several insect species that challenge agriculture and horticulture. In particular, insect species with a wide host range, including several cultivated plants, can cause high economic losses in newly invaded regions [1,2,3]. One insect species that benefits from human activities and worldwide trade through hitchhiking and warmer climatic conditions is the pentatomid *Halyomorpha halys* (Stål, 1855) [4]. The brown marmorated stink bug invaded and established itself successfully in several countries far from its native habitat in Eastern Asia. Since the mid-1990s, it is found in the USA. Since 2007, it started invading Europe and was first detected in Germany in 2011 [5]. In its new habitats, *H. halys* quickly became a serious problem for farmers [6]. As a highly polyphagous insect, *H. halys* feeds on over 300 different plants, including some common plant families such as Rosaceae and Saphindaceae [1]. Of particular agricultural importance are woody perennials such as apple, pear, peach, grape, and hazelnut, as well as annuals like bell pepper, corn, and soy bean [7]. Its piercing–sucking feeding behaviour on these fruiting plants causes abortion or malformation and necrotic areas on feeding sites that make fruit unmarketable [8]. Damage by all developmental stages of the bug, overlapping generations during summer [9], and no effective plant protection strategy can lead to high economic losses, especially in newly invaded regions. In the USA, *H. halys* caused US$37 million losses in apple production in 2010 [10]. In Italy, seven years after its official detection [11], the invasion of *H. halys* led to 80–100% yield losses and estimated economic losses of €588 million in apple production in the year 2019 [12]. These high damages were due to a lack of effective plant protection strategies or natural enemies [1]. In Germany, the only commercially available tools are aggregation pheromone-baited traps for monitoring the bugs. However, they are not suitable for mass trapping of bugs and thus not for reduction in the overall damage to fruit orchards [13].

To develop effective, environmentally friendly strategies for pest management, it is important to understand how insects and plants interact with each other. Despite the broad host range of the bugs, some typical behaviours and preferences are already known. *H. halys* can discriminate among hosts, switches between wild hosts and cultivated plants, and seems to prefer non-Asian over Asian species [14,15,16,17]. It prefers certain hosts over others and requires a diverse diet and fruiting structures to complete its development from egg to adult [18]. The cues responsible for host choice are still unknown, but previous studies highlight plant stimuli, including visual, vibrational, gustatory, thermal, and especially volatile stimuli [16,18,19,20]. Plants develop through different phenological stages, during which both olfactory and visual cues vary [21,22]. These signals also differ between plant species. *H. halys* appears to distinguish between these complex variations to differentiate among hosts, as well as among various phenological stages within a single plant species [23].

For most insects, olfaction is an important sensory modality that enables them to interact with their environment and to locate essential resources, such as hosts, food, or mating partners [24]. In particular, the ability to discriminate between volatile blends that differ in the qualitative and quantitative composition of their compounds is considered crucial for several insect species [25,26,27]. For monophagous insects, like the pear psyllid *Cacopsylla pyri*, a specific mixture of typical host volatiles attracts the insect [28]. Finding volatile compounds that are important for host selection in generalist insects is more challenging than for specialists [29,30]. For *H. halys,* little is known about the specificity of volatile cues involved in host selection. As a first step towards finding volatile compounds potentially important, our objectives were to (1) identify highly attractive phenological stages of host plants in the field, (2) compare the volatile organic compound profiles of the different host plant phenological stages, (3) identify compounds that are perceptible by *H. halys*, and (4) test the behavioural responses of the bugs when exposed to single compounds and mixtures.

## 2. Materials and Methods

### 2.1. Insect Rearing

Wild *H. halys* were collected in the field from May to August 2023. Bugs hatched from eggs laid by these field-collected individuals were used to establish a laboratory colony. The colony was maintained under controlled conditions in a climate chamber at 24 °C, 70% relative humidity, and 16:8 h (L:D) photoperiod. Adults were reared in cages (30 cm × 30 cm × 30 cm; BugDorm, MegaView Science, Taichung, Taiwan) with a sex ratio of 1:1 (male:female) and fed ad libitum with fresh green beans, tomatoes, and sunflower seeds. Food was replaced and eggs were collected twice a week. A plastic container filled with water and a sponge cloth provided drinking water. For behavioural experiments, 2–3 week-old reproductively mature females were used.

### 2.2. Chemicals

Synthetic standard chemicals used for bioassays and electrophysiological tests were dissolved in n-hexane (>99%, Sigma-Aldrich, St. Louis, MO, USA), which served as a solvent. The tested compounds included (*E*)-2-decenal, *cis*-3-hexenyl acetate, decanal, hexanal, octanal, (*E*)-4,8-dimethyl-1,3,7-nonatriene (DMNT), geranyl acetone, and 6-methyl-5-hepten-2-one (all obtained from Merck KGaA, Darmstadt, Germany).

### 2.3. Population Monitoring

Monitoring was conducted from July 2023 to July 2024 in commercial orchards in Hirschberg, Germany. At these sites, the orchards were managed under integrated plant protection and had been completely harvested. The study encompassed two monoculture apple and plum orchards, one monoculture pear orchard, and one mixed-species orchard with quinces. All sample sites were located within an agricultural landscape, surrounded by other perennial and annual crops, as well as some hedges, with only isolated buildings nearby (e.g., farmsteads), approximately 1 km from the outskirts of the town. The quince orchard was located on the outskirts of the town and was surrounded by other perennial crops, hedges, and buildings. Four traps were deployed in each of the larger monoculture orchards (apple, plum, and pear), while two traps were installed in the smaller mixed-species quince orchard. A second monitoring phase took place from May to November 2024 at the experimental field station of the Julius Kühn Institute (JKI) in Dossenheim, Germany. At this site, integrated pest management was applied, and the cherry orchards were left unharvested. Moreover, the field station consists of diverse fruit crops, a building complex, as well as border hedges and other annual agricultural crops. During this second monitoring phase, aggregation pheromone baited traps were deployed as follows: six in apple orchards, three each in cherry and pear orchards, four in quince orchards, and three in a mixed stone fruit field (apricot, plum, peach). Since there were no commercial cherry orchards available for bug sampling nearby, cherry trap catches from the experimental field were also used for the 2023 monitoring. Commercial traps (Trifolio-M GmbH, Lahnau, Germany) baited with a dual lure combining an aggregation pheromone and a synergist (Pherocon^®^, Trécé Inc., Adair, OK, USA) were installed in the trees at approximately 1.50 m above ground. The required minimum distance between the traps within an orchard was 25 m. The number of traps (see Table A1) was adjusted according to the size of the orchard and the distance between traps. Pheromone dispensers were replaced every 12 weeks. Weekly counts of trapped adult bugs and nymphs were conducted, recording sex and nymphal stages. Furthermore, plant growth developmental stages at times of high bug abundances were recorded based on the principal growth stages (BBCH) described by Meier [31].

### 2.4. Volatile Analysis

#### 2.4.1. Headspace-Sampling

Headspace samples were taken in situ from apple (*Malus domestica* cv. Braeburn; BBCH 87/89), pear (*Pyrus communis* cv. Williams Christ; BBCH 87/89), quince (*Cydonia oblonga;* BBCH 74), plum (*Prunus domestica* cv. Franzi; BBCH 76), peach (*Prunus persica* cv. Benedicte; BBCH 87/89) and cherry (*Prunus avium* cv. Schneider’s Späte Knorpelkirsche; BBCH 85). At these stages, a higher abundance of bugs was observed on the respective fruit crop compared to the other fruit crops during the same sampling week. The sampling procedure used was previously described [32,33]. Single intact twigs with fruit and leaves that appeared healthy and were not infested were carefully wrapped in plastic oven bags (Toppits^®^, Melitta, Minden, Germany). A purified airstream (1 L/min) controlled by a headspace collecting device [33] was pumped through teflon tubing into the bag to a final volume of 30 L. Samples were trapped on stainless steel, prepacked sample tubes with Tenax^®^ TA35/60 sorbent (Markes International GmbH, Offenbach, Germany). Prior to use, oven bags were heated for at least 2 h at 60 °C, and teflon tubing was rinsed with 70% ethanol and baked for 2 h at 120 °C. Both materials were equilibrated to ambient temperature before sampling. Headspace from apple (*n* = 6), cherry (*n* = 11), peach (*n* = 10), pear (*n* = 9), plum (*n* = 9), and quince (*n* = 10) were sampled and analysed.

#### 2.4.2. Thermodesorption-GC-MS

For chemical analysis of the collected headspace samples, an automated thermal desorption system (TurboMatrix™ ATD 650, PerkinElmer, Rodgau, Germany) connected to a gas chromatograph coupled with a mass spectrometer (TD-GC-MS) was used. Desorption of sampling tubes was 10 min at 250 °C, cold trap was held at −20 °C and then heated at 99 K/s to 250 °C and 1 min desorption time. For GC separation of volatile compounds, a non-polar Elite-5MS (Crossbond 5% diphenyl/95% dimethyl polysiloxane, PerkinElmer) capillary column (30 m × 0.25 mm id × 0.25-μm film thickness) was used. Helium (5.0, Linde, Munich, Germany) with a column head pressure of 130 kPa was used as carrier gas. The GC programme was as follows: oven temperature was kept at 40 °C for 1 min, then heated from 50 to 180 °C at 5 K/min and finally increased to the final temperature of 280 °C by a rate of 20 K min/min and held at 280 °C for 6 min. The GC inlet line temperature was 250 °C, and the ion source temperature was set to 180 °C. The quadrupole mass detector operated at 70 eV ionization energy and the full-scan mass spectra were within the range of 35–350 *m*/*z*.

#### 2.4.3. Identification and Quantification with AMDIS

Chromatograms of collected volatile samples were analysed using the “Automated Mass spectral Deconvolution and Identification System” (AMDIS, V. 2.71; National Institute of Standards and Technology NIST, Boulder, CO, USA). The identification procedure followed Gross et al. [32]. Ion fragmentation patterns and retention indices were compared with those of standard compounds. Identification criteria were set as follows: a match factor ≥ 85% and a relative retention index deviation ≤ 3% from the reference value. Deconvolution settings were as follows: component width: 32; adjacent peak subtraction: one; resolution: low; sensitivity: very low; shape requirements: low; level: very strong; maximum penalty: 20; “no RI in library”: 20. Compounds that could not be identified were listed as unknowns. Components with a signal-to-noise ratio < 100 were excluded from the analysis.

### 2.5. Electroantennography (EAG)

The electrophysiological responses of the *H. halys* adult antennae to synthetic volatiles were tested using the following procedure: Antennae were excised at the base of the scape and immediately mounted on an antenna holder (PRG-2, Ockenfels Syntech GmbH, Buchenbach, Germany) with electrode gel (Signa gel; Parker Labs, Farfield, NJ, USA). The antenna holder was connected to an EAG combi-probe with internal gain 10× (Ockenfels Syntech GmbH) and placed at the end of a glass tube where a charcoal-filtered and humidified airstream (1.0 L/min) passed. Synthetic volatiles were diluted in n-hexane to a concentration of 100 µg/µL and tested randomly. An aliquot of 1 µL was pipetted onto a filter paper (Type 413, VWR Collection, Radnor, PA, USA) and placed in a glass Pasteur pipette. The solvent n-hexane served as a control. Each experiment started and ended with a puff of 100 µg (*E*)-2-decenal as a positive control [34] to confirm that the antenna elicited reproducible and reliable responses. Stimulations with a flow of 0.5 L/min and a duration of 1 s were manually applied by a pedal switch connected to a data acquisition controller (IDAC-2, Ockenfels Syntech GmbH). Intervals between stimuli were 1 min. For each compound, the electrophysiological response from ten females and males, and in the case of nonanal and DMNT, six males, was recorded with EAGPro software (version 1.1, Ockenfels Syntech GmbH) and used for analysis.

### 2.6. Olfactometer Assay

For olfactory experiments, a custom-made dynamic Y-shaped glass olfactometer (Glasgerätebau OCHS Laborfachhandel e.K., Bovenden, Germany) was used. The entrance arm was 195 mm long, while the test arms were 180 mm long and had an angle of 50°. The tubing had a diameter of 40 mm. Experiments were conducted at room temperature (23 °C) in a photo box (60 × 60 × 60 cm; GODOX Photo Equipment Co., Ltd., Shenzhen, China) and a dark room to exclude visual stimuli. The lighting strips (5800 K; 12,000 lumen) belonging to the photo box were attached to the left and right sides of the photo box 36–40 cm above the olfactometer. A paper, size A3, printed with 2 cm wide black-and-white horizontal stripes was placed under the olfactometer to aid orientation of the moving test insect. A charcoal-filtered airstream, set to a flow of 75 mL/min provided by a clean air delivery system (CADS-4CPP-variable humidity, Sigma Scientific LLC, Micanopy, FL, USA), was humidified and pumped through the odour source chamber connected to the test arms. Experiments were conducted with females only, as female orientation behaviour towards olfactory cues is directly linked to host plant selection and oviposition, whereas male behaviour is often dominated by reproductive motivation. Bugs were starved 24 h before the experiments, then single females were gently placed in front of the entrance arm and allowed to enter the tube deliberately. A choice was made when the insect reached more than two-thirds of the arm. Bugs that did not reach the test arms within 5 min were recorded as “no choice”. After five replicates, the system was rotated to avoid positional bias. All tubes, the odour source chamber, and the olfactometer were rinsed with 70% EtOH and baked at 100 °C for two hours after the tests. Each insect was tested only once.

As single synthetic volatiles, hexanal, DMNT, *cis*-3-hexenyl acetate, and 6-methyl-5-hepten-2- one were tested in amounts of 1 µg or 100 µg. Hexanal and DMNT were additionally investigated at a dose of 10 µg. Those four compounds were found to be the main components of the volatile blend across the examined plant taxa and were not classified as repellent in other olfactory studies [35]. Furthermore, a potentially attractive mixture (paM; Table 1) was tested undiluted in a 1:10, a 1:100, and a 1:500 dilution. The composition of the paM reflected the overall mean relative proportions of volatiles that contributed most to the odour profile of all sampled fruit crops (Table A2). Based on the results of a previous behavioural study with nymphs, which showed avoidance to octanal and decanal and an indifferent behaviour to nonanal [35], we also excluded these aldehydes from the paM. All compounds were pipetted onto pieces of filter paper and then placed in the odour chamber.

### 2.7. Statistics

All statistical analyses were performed in R version 4.3.1 “Beagle Scouts” [36]. The R package ggplot2, version 4.0.0, [37] was used for graphical depiction of the results.

#### 2.7.1. Volatile Analyses

A compositional data set was calculated for investigating the volatile profiles of the different host plants. All components that appeared less than three times in the whole data set were excluded. Permutational multivariate analysis of variance PERMANOVA [38] was used as a multivariate test for discrimination of groups. To ensure that the significant PERMANOVA result is based on location and not on dispersion effects, a permutational analysis on multivariate dispersions PERMDISP [39] was performed to test for homogeneity of multivariate dispersion. Both tests were conducted with Bray–Curtis dissimilarities [40] and the R package vegan version 2.6-6.1 [41]. For pairwise comparisons between the volatile profiles of the different host plants, permutation MANOVAs with a BH *p*-value adjustment method were used with the R package RVAideMemoire version 0.9-83-7 [42].

#### 2.7.2. EAG Recordings

The Wilcoxon matched pairs signed-rank test was used to detect significant differences between EAG signals in response to the test compound and the respective solvent control. The absolute EAG response (mV) to each test stimulus was subtracted by the response to the solvent controls in order to compensate for solvent and mechanosensory artefacts [43].

#### 2.7.3. Olfactometer Assay

Preference for a volatile or a mixture of volatile compounds was evaluated with a binomial test. Only insects that made a choice were considered in the analysis.

## 3. Results

### 3.1. Monitoring

**Figure 1:**
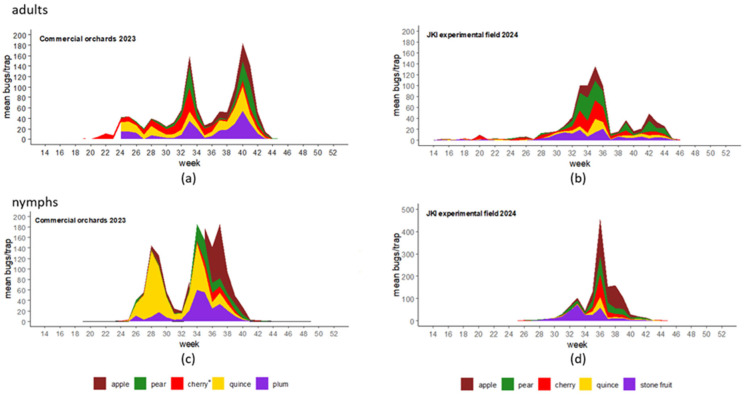
Stacked area graph of captured *H. halys* per trap and week in different fruit trees. Traps were placed at commercial orchards in Hirschberg (2023) (**a**,**c**) and at the experimental field at the Julius Kühn Institute (JKI) in Dossenheim, Germany (2024) (**b**,**d**). Monitoring data were collected weekly from April to November, and the number of adults (**a**,**b**) and nymphs (**c**,**d**) was counted. * Data taken from traps at the experimental field of the JKI, Dossenheim (2023).

Two complete generations of *H. halys* were observed during our monitoring at all sites in the years 2023 and 2024 (Figure 1). For the year 2023, the peak for first-generation adults was reached in week 33; for the second generation, it was observed in week 40. In 2024, the first generation of adult bugs peaked in week 35, while the second generation peaked in week 42. The highest abundances of the bugs were observed depending on the chronological order of fruit maturation in both years. Cherry, the first mature fruit, was always the first crop with high abundances of adult bugs (Figure 1a,b), then the other stone fruits, plum and peach, followed. *H. halys* nymphs were first found to be most abundant on plum and quince (Figure 1c) or the mixed stone fruit area at the experimental field station (Figure 1d). Quince also had a high abundance of adult bugs and nymphs early in summer 2023 (Figure 1a,c). This was not observed for the year 2024 at the experimental field. Pome fruits, such as apple and pear, that mature later in the year showed high numbers of *H. halys* adults and nymphs in late summer and early autumn (from week 33 onwards) (Figure 1). The following highly attractive phenological stages were detected: BBCH 74 for quince (week 27), BBCH 76 for plum (week 24), BBCH 85 for cherry (week 23), and BBCH 87/89 for apple, pear, and peach (week 36, week 33, and 31, respectively).

### 3.2. Volatile Analysis

**Figure 2:**
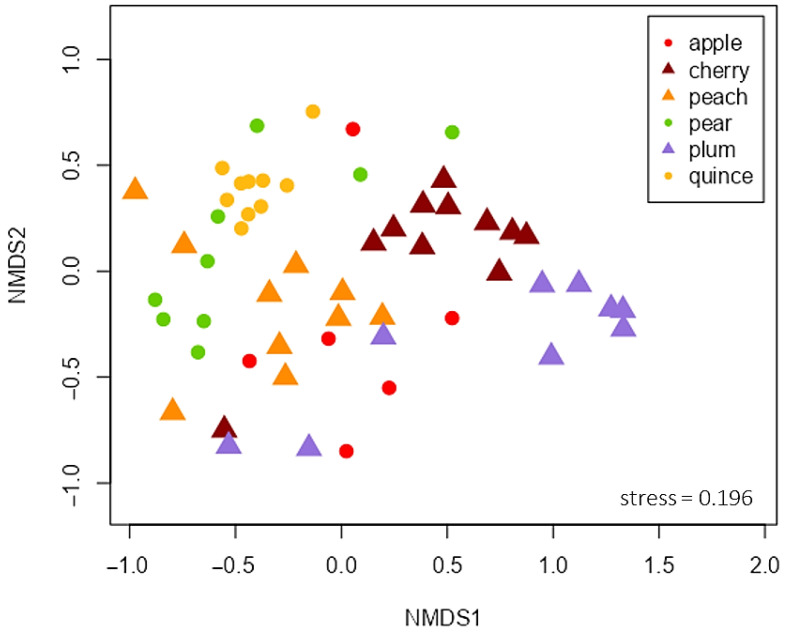
Non-metric multidimensional scaling (NMDS) plot visualizing dissimilarities (Bray–Curtis) of the single volatile profiles (130 substances) of pome fruit (apple BBCH 87/89 (*n* = 6), pear BBCH 87/89 (*n* = 9), quince BBCH 74 (*n* = 10)) are visualized as circles, profiles of stone fruit were depicted as triangles (cherry BBCH 85 (*n* = 11), peach BBCH 87/89 (*n* = 10), plum BBCH 76 (*n* = 9)).

At total of 130 different volatiles were detected over all samples; 79 of them were identified (Table A2). The volatile profile of the different plant species varied significantly (PERMANOVA, df = 5, F = 9.97, *p* < 0.001; Table 2) (Figure 2). This difference explained 50.44% of the overall variance among sampled fruit trees, while the variability within the volatile pattern did not differ significantly between the species (PERMDISP, df = 5, F = 1.25, *p* = 0.31).

**Figure 3:**
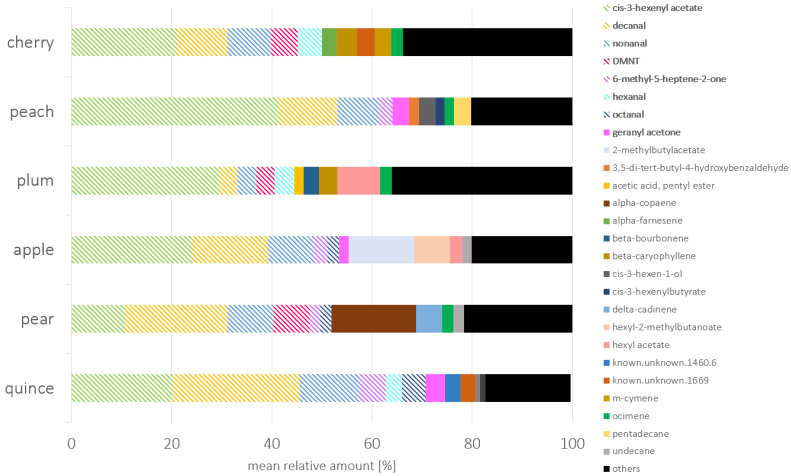
Relative amount of the ten main compounds of the volatile profiles of stone fruit (cherry, peach, plum) and pome fruit (apple, pear, quince). Compounds that were present in high amounts in all plant species were marked with dashed lines. Compounds tested in EAG or olfactometer were printed in bold.

The dominant volatile organic compound across all species was *cis*-3-hexenyl acetate, a green leaf volatile (GLV), which accounted for between 11% (pear) and 41% (peach) of the total relative composition of the volatile profile among the different hosts (Figure 2). The aldehydes decanal and nonanal constituted a high proportion of the volatile profile across all hosts. Decanal was also the main compound for quince (25%) and pear (20%). Octanal was detected in the headspace of all hosts, ranging from 0.5% (plum) to 4.7% in quince. All host plants emitted DMNT and 6-methyl-5-hepten-2-one in relatively high amounts, varying from 0.62% (apple) to 7.20% (pear) for DMNT and 0.4% (plum) to 5.51% (quince) for 6-methyl-5-hepten-2-one (Figure 3). Hexanal was found in all samples except from peach. Apple and pear trees emitted lower amounts of this aldehyde, with hexanal accounting for a moderate relative proportion of 0.8% (apple) and 0.5% (pear) of their volatile profile. Additionally, compounds with relatively high proportions were 2-methylbutyl acetate (13%) and hexyl-2-methylbutanoate (7%) for apple samples, α-copaene (17%) for pear samples, and β-caryophyllene (4% and 3.7%) for cherry and plum samples, respectively. For peach trees, *cis*-3-hexenol was found in higher relative amounts (3.3%) compared to the other species.

### 3.3. Electroantennography

**Figure 4:**
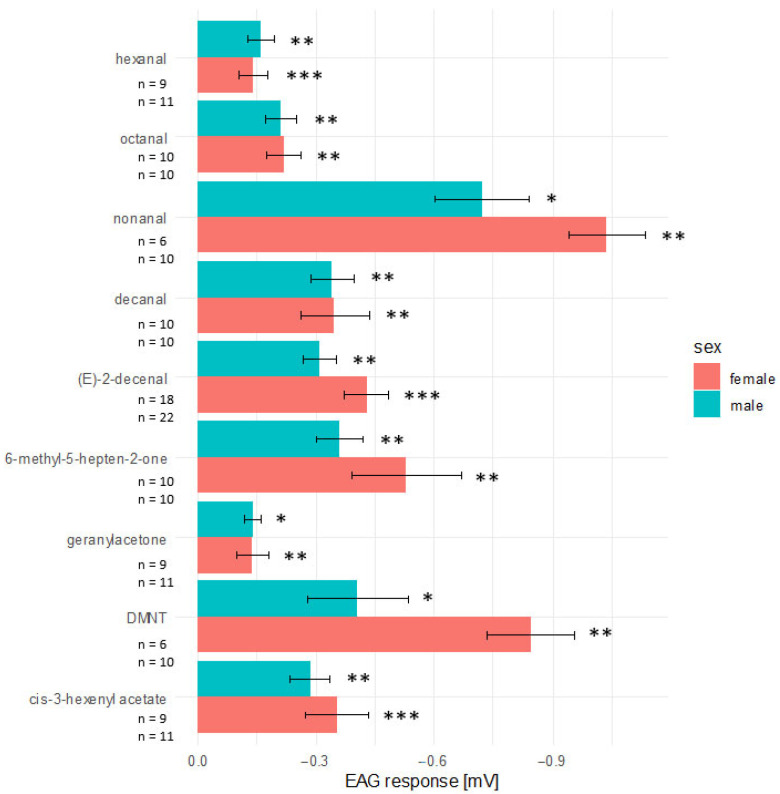
Mean ± standard error of the summated receptor potentials of male and female bugs in response to an odour puff of 100 µg of synthetic volatiles. Responses to the solvent control were subtracted. Significant responses compared to solvent control were marked with asterisks (Wilcoxon matched pairs signed-rank test, *, *p* < 0.05, **; *p* < 0.01; ***, *p* < 0.001).

The following criteria were used to select compounds for electroantennography: (a) compounds had to account for a large mean proportion of the volatile profiles across the host plants, (b) they needed to be present in at least five of the six plants (Figure 3), and (c) rank among the 10 most abundant compounds in at least three of the sampled fruit crops. A puff of 100 µg (*E*)-2-decenal applied on the antenna of male and female bugs served as a positive control and elicited a reliable and significantly stronger reaction compared with the solvent control (Wilcoxon matched pairs signed-rank test, male: V = 153.5, *p* < 0.01; female: V = 0, *p* < 0.001) (Figure 4). For *cis*-3-hexenyl acetate, the antenna of both sexes showed significant antennal responses (Wilcoxon matched pairs signed-rank test; male: V = 0, *p* < 0.01; female: V = 0, *p* < 0.001) (Figure 4). A puff onto the antenna with the single aldehydes decanal, hexanal, nonanal, and octanal led also to significant reactions in females and males (Wilcoxon matched pairs signed-rank test, decanal: male: V = 0, *p* < 0.01; female: V = 0, *p* < 0.01; hexanal: male: V = 0, *p* < 0.01; female: V = 0, *p* < 0.001; nonanal: male: V = 0, *p* < 0.05; female: V = 0, *p* < 0.01; octanal: male: V = 0, *p* < 0.01; female: V = 0, *p* < 0.01) (Figure 4). The same was found for DMNT (Wilcoxon matched pairs signed-rank test, male: V = 0, *p* < 0.05; female: V = 0, *p* < 0.01), 6-methyl-5-hepten-2-one (Wilcoxon matched pairs signed-rank test, male: V = 0, *p* < 0.01; female: V = 0, *p* < 0.01), and for geranyl acetone (Wilcoxon matched pairs signed-rank test, male: V = 0, *p* < 0.05; female: V = 0, *p* < 0.01) (Figure 4).

### 3.4. Olfactometer Assay

**Figure 5:**
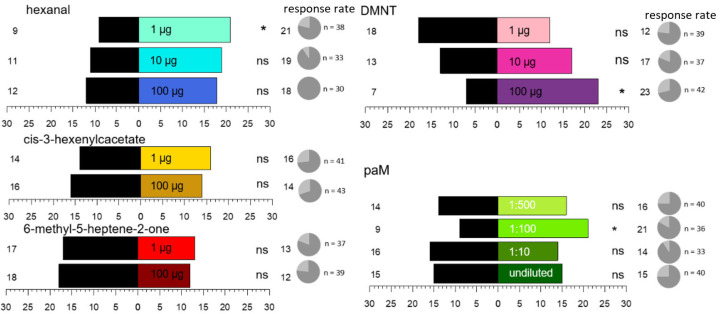
Choice of female *H. halys* adults (*n* = 30) for single synthetic VOCs and a potentially attractive mixture (paM) (coloured) against solvent control (black) in a Y-tube olfactometer (*, *p* < 0.05; ns = not significant, binomial test). Percentage of bugs that made a choice (dark grey) and that did not (light grey) is presented as response rate as pie charts on the right. N gives the total number of tested bugs per treatment.

Four compounds were tested in the Y-tube olfactometer, both individually and as a mixture. Among the individual synthetic volatiles examined, female *H. halys* showed a significant preference for 100 µg DMNT and 1 µg hexanal (binomial test, *p* = 0.04) (Figure 5). For the other test compounds, the bugs did not show a significant preference or avoidance (binomial test, *p* > 0.05) (Figure 5). For the synthetic mixture of main compounds of the host plant volatiles, the bugs showed an attraction to the 1:100 dilution (binomial test, *p* = 0.04). Motivation of the insects was consistently high throughout these assays, with at least 70% making a choice for one of the sides of the olfactometer (Figure 5).

## 4. Discussion

In the present study, we monitored the abundance of *H. halys* throughout the season, comparing a range of commercial fruit crops. The volatile compounds emitted by these plants were sampled and analysed at the time point of high bug abundances. Electrophysiological responses to single compounds and behavioural reactions to both individual compounds and a synthetic volatile blend were examined.

Different abundances of bugs were observed among host plants throughout the fruiting season. Also, the volatile patterns of the various crops differed significantly at times of highest abundance of the insects. However, some compounds were common across the investigated plant species. *Halyomorpha halys* perceived those typical plant volatiles via their antennae, but showed a significant attraction in the Y-olfactometer only to hexanal and a higher concentration of DMNT (100 µg). For hexanal, a similar attraction was observed for all tested doses, but was significant only at a dose of 1 µg. This indicated a clear and consistent attraction to this volatile compound, with only weak dose-dependent effects observed at higher concentrations. The observed divergence between antennal sensitivity and behavioural output underscores that odour detection at the peripheral sensory level does not necessarily result in attraction or repellency. Instead, behavioural responses likely reflect central integration of olfactory signals and their ecological relevance, highlighting that electrophysiological responsiveness alone is insufficient to predict behavioural outcomes. Trap captures varied over the sampling years, with higher numbers generally coinciding with the seasonal presence of host plants and fruit at specific phenological stages. This finding is supported by other studies which proposed that there is a close relationship between plant phenology, host quality, and presence of *H. halys* [15,17,18,23,44,45]. Acebes-Doria et al. [46] observed seasonal trends in the movement of *H. halys* nymphs among apple and peach trees, as well as wild host plants. A study by Acebes-Doria et al. [18] also showed that polyphagy and access to multiple host plants can improve fitness and survival of *H. halys*. Since host switching plays an important role in this context, our two-year, multi-host monitoring across different fruit crops provides new insights into the seasonal abundance patterns of the bugs. The high dispersal capacity of adult bugs and their movement patterns depend on different factors, such as host distribution and availability, as well as on ecosystem features [47]. Nymphs appear to be more strongly influenced by the composition of habitats in the agricultural landscape. These effects cannot be observed in adult bugs, which are instead influenced by the fruit crops grown [48]. Our monitoring was conducted in similar agricultural landscapes, and showed differences in bug abundances among different fruit crops over the season.

Bearing fruits, especially mature fruits, strongly influences the host choice of the bug and can cause high abundances [49]. This is what we have also seen during our two-year monitoring. The highest bug abundances were generally observed on hosts bearing ripe or more advanced-stage fruits compared to other host plants. This also explains the order of host preferences over the season. The first ripening fruits of the season can be found on stone fruit trees, beginning with cherry, followed by peach and plum. Pome fruit ripens later in the year, around August and September, depending on the variety, and *H. halys* were more abundant on these hosts later in the season. Especially for apple and pear trees, weekly captures per trap increased in late summer and reached their peak when the fruit was ripe for harvest. This late occurrence of the stink bugs, with a peak before the harvest of the fruit, was also shown for pear and apple in the Mid-Atlantic in the United States [50]. A possible reason for the early high number of bugs in quince 2023 could be the large fruit size compared to the other crops, or it might be influenced by the proximity to peach trees in this orchard. The traps hung in the cherry orchard showed high abundances for the whole summer, which might have been caused by the fact that those cherries were not harvested in 2023 and 2024. The fruit mummies were still on the trees until late autumn and could provide a long-lasting food source for the bugs before they searched for sheltered overwintering sites and entered reproductive diapause.

The volatile profiles of the different host plants varied significantly in their composition. Some plants emitted a larger number of volatile compounds, such as apple, cherry, and plum, while other emitted fewer, including peach, pear, and quince. The main compounds of the volatile bouquet found across all examined host plants were typical plant volatiles, which were also found in other studies on *H. halys* host plants [23,51,52,53,54,55].

The polyphagous feeding pattern of the stink bug, and thus its broad host range with varying volatile profiles, might explain the results of the EAG, where all tested volatiles elicited significant antennal responses. While specialists only need to perceive host-specific compounds to find their host, for polyphagous insects, several volatiles could be important. Thus, for generalists, it is more difficult to select the candidate substances from the specific profile that affect the behaviour of the insects compared to specialists [29,30]. This is in accordance with Bernays’ Neural Limitation Hypothesis [56], which suggests that specialist herbivores have evolved a neural sensitivity and only perceive specific volatiles. In contrast, generalists are sensitive to a wider range of broadly occurring chemical signals.

One of these widespread chemicals is *cis*-3-hexenyl acetate. This compound comprised a large proportion of the total volatile content in almost all collected samples. It belongs to the group of green leaf volatiles, which are ubiquitous odorants among several plant species [57], and can thus be an olfactory cue for a generalist stink bug. Investigations on GLVS are a frequently used approach to decipher host attractiveness to *H. halys* and have already been shown to increase the retention time of the bug on host plants [20]. As a single component, *cis*-3-hexenyl acetate has not led to a significant behavioural response besides its perception through the bug. Similar results were reported for *H. halys* nymphs in earlier studies [35].

To identify a general olfactory signal that enhances the bugs’ host choice, a synthetic blend of the four main volatiles of all studied host plants was tested. A mixture of compounds that are common across different plant taxa often plays a role in the host-finding process of phytophagous insects [58,59]. For this reason, a mixture of common volatiles across the selected species was developed based on our results. Among the single compounds of this blend, we found a significant preference for hexanal and DMNT. The aldehyde hexanal, a compound also emitted by fresh peanut seeds, is known to elicit foraging and proboscis-protruding behaviour of *H. halys* [60]. DMNT is found in high amounts in blooming sunflowers, the phenological stage preferred by female bugs, and is part of an attractive synthetic sunflower volatile mixture [23]. Adding this volatile mixture containing DMNT to pheromone baited traps increased trap captures of *H. halys* in early spring [23]. Furthermore, the bug species *Apolygus lucorum* showed a preference for high emissions of DMNT from its host [61].

A synergistic effect from the other compounds of the mixture could not be observed in the Y-shaped olfactometer, indicating that DMNT and hexanal alone might have affected the insect’s behaviour. However, olfactometer bioassays have their limitations, especially for insects that rely on cues other than chemical ones for orientation and host finding. Long-range foraging in insects is generally guided by semiochemicals [26]. Despite the artificial test environment of an olfactometer, the effects of these semiochemicals can be tested and evaluated in a standardized in a simplified manner. However, it must be considered that short-range orientation can be mediated by factors such as the size and shape of plants, colours of host plants and fruit [62,63], and polarized light reflections from the foliage [64]. These cues are excluded in olfactometer assays. The study by Wong et al. [23], in which the settling choices of the bugs did not align with the results of the olfactometer assay, suggested the influence of such cues. It is already known that visual stimuli and gustatory cues are involved in pentatomids’ host plant selection and orientation. For *H. halys,* visual stimuli in pyramid traps provide orientation [65]. Furthermore, intensity and wavelength of light affect the behaviour of the bugs [66,67]. An overview of traps combining olfactory and visual stimuli is reviewed by Weber et al. [68]. These signals could also contribute to host selection and should be further investigated. Moreover, *H. halys* is known to use vibrational signals to mediate mating behaviour. Artificial vibrations mimicking female calling patterns can attract *H. halys* and improve the efficacy of pheromone traps [69,70]. In addition, host probing is a typical behaviour of this species [71] and cannot be assessed in olfactometer tests. Traps equipped with the male-produced aggregation pheromone, a 3.5:1 mixture of two stereoisomers, (3*S*,6*S*,7*R*,10*S*)-10,11-epoxy-1-bis-abolen-3-ol and (3*R*,6*S*,7*R*,10*S*)-10,11-epoxy-1-bisabolen-3-ol [72], trapped many individuals (males, females, and nymphs), while also attracting more bugs to the orchards around the traps. Adding another stink bug aggregation pheromone, methyl (*E,E,Z*)-2,4,6-decatrienoate from *Plautia stali*, enhances the attraction of *H. halys* [73]. Thus, the pheromone and this synergist alone cannot be used for mass trapping of the insect [13]. By combining the pheromone and synergist with a host plant-derived kairomone, further synergistic effects might increase the effectiveness of a trap by enhancing the short-distance attraction. Furthermore, combining chemical (kairomones and pheromones), visual, and vibrational stimuli in one trap could be a sustainable solution for future control of *H. halys*.

## 5. Conclusions

For developing new, effective strategies for plant protection, it is important to gain a better understanding of host selection and the driving factors of host choice. Our study is the first to simultaneously examine the abundance of *H. halys* on various commercial fruit crops, to identify attractive phenological plant stages, to examine the volatile profiles of these plants, and to determine the semiochemical similarities among the hosts. Despite the broad range of perceived volatiles, only hexanal and DMNT were attractive to *H. halys*. This integrated approach provides a broader ecological understanding of host–insect interactions and offers a basis for developing plant-based attractants for use in integrated pest management and organic systems.

## Figures and Tables

**Table 1:** Composition of the synthetic produced potentially attractive mixture (paM).

Chemical	Proportion [%]	Mass Concentration mg/mL
*cis*-3-hexenyl acetate	24	215.28
DMNT ^1^	3	24.00
6-methyl-5-hepten-2-one	2.5	21.10
hexanal	2.2	17.57

^1^ (*E*)-4,8-dimethyl-1,3,7-nonatriene.

**Table 2:** Results of pairwise comparisons of the volatile pattern of the different host plants using permutation MANOVAs based on Bray–Curtis dissimilarities of different host plants. **, *p* < 0.01; ***, *p* < 0.001.

	Cherry	Peach	Pear	Plum	Quince
apple	***	***	***	***	**
cherry		***	***	***	***
peach			***	***	***
pear				***	***
plum					***

N (permutations) = 10,000; *p*-adjustment method: BH.

## Data Availability

The original contributions presented in this study are included in the article. Further inquiries can be directed to the corresponding author.

## Abbreviations

The following abbreviations are used in this manuscript:

DMNT	(*E*)-4,8-dimethyl-1,3,7-nonatriene
EAG	Electroantennography
GC-MS	Gas chromatography–mass spectrometry
GLV	Green leaf volatile
JKI	Julius Kühn Institute
TD	Thermodesorption
VOC	Volatile organic compound

## Appendix A

**Table A1:** Overview over monitoring traps baited with a dual lure.

	Commercial Orchard (Hirschberg) July 2023–November 2023		Experimental Field Station (JKI) May 2024–November 2024	
	No. of Fields	Traps Per Field (Total Number of Traps)	No. of Fields	Traps Per Field (Total Number of Traps)
apple	2	4 (8)	2	3 (6)
cherry	-	-	1	3
quince	1	2	2	2 (4)
pear	1	4	1	3
plum	2	4 (8)	-	-
Other stone fruit ^1^	-	-	1	3

^1^ including peach, plum, and apricot.

## Appendix B

**Table A2:** Retention indices and arithmetic mean of the relative proportions of volatile compounds included in the analysis, detected in headspace samples of apple, cherry, peach, pear, plum, and quince. The last column shows the overall arithmetic mean of each volatile across all fruit crop species. For visualization, a heatmap showing low-contributing components (green), medium-contributing components (orange), and high-contributing components (red) is added.

Volatile	RI	Apple	Cherry	Peach	Pear	Plum	Quince	Mean
***cis*** **-3-hexenyl acetate ***	1005	24.19	21.10	41.41	10.67	29.61	20.26	24.54
**decanal ***	1205	15.03	10.05	11.62	20.46	3.55	25.29	14.33
**nonanal ***	1105	9.01	8.72	8.27	9.18	3.71	11.73	8.44
**DMNT ***	1094	0.62	5.27	1.48	7.20	3.69	0.34	3.10
α-copaene	1374	0.00	0.00	0.00	16.89	0.65	0.00	2.92
**6-methyl-5-heptene-2-one ***	986	2.82	1.88	2.74	2.18	0.40	5.51	2.59
**hexanal ***	802	0.83	4.87	0.00	0.54	3.90	3.17	2.22
2-methylbutyl acetate *	870	13.07	0.00	0.00	0.00	0.00	0.00	2.18
**octanal ***	1001	2.34	2.30	0.81	2.20	0.46	4.73	2.14
**geranyl acetone ***	1454	1.93	1.13	3.34	1.47	0.44	3.90	2.03
hexylacetate *	1012	2.50	0.68	0.42	0.00	8.51	0.00	2.02
undecane	1100	1.95	1.31	0.90	2.13	1.62	2.09	1.67
known.unknown.1669	1669	0.05	3.56	1.20	0.69	0.95	2.96	1.57
β-caryophyllene *	1420	0.00	4.00	1.02	0.42	3.74	0.00	1.53
ocimene *	1042	0.07	2.31	1.81	2.27	2.28	0.12	1.48
pentadecane	1500	0.45	0.73	3.41	2.10	1.22	0.00	1.32
hexyl-2-methylbutanoate *	1236	7.10	0.00	0.00	0.00	0.00	0.00	1.18
α-farnesene	1507	0.75	2.95	0.44	0.84	1.63	0.00	1.10
known.unknown.1460.6	1461	0.32	0.24	0.76	1.30	0.48	3.06	1.03
methyl salicylate *	1193	0.57	1.06	0.29	1.03	1.55	1.26	0.96
3,5-di-tert-butyl-4-hydroxybenzaldehyde	1774	0.00	0.82	2.00	0.07	0.80	1.94	0.94
δ-cadinene	1530	0.00	0.00	0.14	5.17	0.00	0.00	0.89
benzaldehyde *	961	0.22	1.11	1.31	0.15	0.37	2.00	0.86
*cis*-3-hexen-1-ol *	858	0.37	0.57	3.26	0.00	0.00	0.28	0.75
known.unknown.852	852	0.11	1.38	0.00	0.53	0.00	2.41	0.74
undecanal *	1308	0.73	0.65	0.00	1.01	0.15	1.52	0.67
m-cymene	1026	0.00	3.30	0.03	0.00	0.28	0.00	0.60
β-bourbonene	1385	0.00	0.10	0.10	0.17	3.06	0.00	0.57
*cis*-3-hexenylbutyrate *	1186	0.21	0.30	1.85	0.00	0.38	0.00	0.46
known.unknown.1193.2	1193	0.00	0.64	1.35	0.00	0.36	0.09	0.41
acetic acid, pentyl ester *	915	0.57	0.00	0.00	0.00	1.86	0.00	0.40
acetic acid, butyl ester *	809	1.82	0.00	0.48	0.00	0.10	0.00	0.40
known.unknown.1354.9	1355	0.00	0.00	0.00	0.75	1.61	0.00	0.39
benzoic acid	1174	0.12	1.31	0.00	0.28	0.11	0.44	0.38
p-xylene *	863	1.40	0.00	0.69	0.00	0.13	0.00	0.37
known.unknown.1503.9	1504	0.00	0.00	0.09	1.97	0.16	0.00	0.37
known.unknown.901.4	901	0.81	0.12	0.16	0.29	0.50	0.30	0.36
3-hexenal	797	0.24	1.10	0.27	0.00	0.00	0.55	0.36
2-pentadecanone, 6,10,14-trimethyl-	1850	0.00	0.40	0.12	0.09	1.13	0.28	0.34
heptadecane	1700	0.20	0.10	0.91	0.37	0.34	0.00	0.32
known.unknown.1459.9	1460	0.00	1.06	0.00	0.00	0.73	0.00	0.30
known.unknown.954.6	955	0.28	0.90	0.00	0.21	0.00	0.39	0.30
acetophenone *	1065	0.39	0.51	0.00	0.44	0.25	0.16	0.29
tridecan	1300	0.53	0.15	0.20	0.30	0.52	0.00	0.28
heptanal*	904	0.12	0.97	0.00	0.00	0.17	0.43	0.28
δ-elemene	1330	0.00	0.00	1.69	0.00	0.00	0.00	0.28
sabina ketone	1160	0.00	0.53	0.72	0.00	0.38	0.00	0.27
*trans*-2-hexenal *	854	0.00	0.96	0.00	0.00	0.00	0.66	0.27
tetradecane	1400	0.16	0.28	0.14	0.38	0.62	0.00	0.26
butylated hydroxytoluene	1518	0.24	0.05	0.00	0.00	0.03	1.22	0.26
known.unknown.1275	1275	0.63	0.61	0.00	0.00	0.02	0.28	0.26
decane	1000	0.54	0.10	0.19	0.16	0.52	0.00	0.25
4-hexen-1-ol, acetate	1008	0.42	0.00	0.00	0.11	0.78	0.16	0.24
1-hexadecene	1563	0.00	0.09	0.00	0.56	0.82	0.00	0.24
styrene	870	0.00	0.00	1.46	0.00	0.00	0.00	0.24
known.unknown.1170	1170	0.48	0.12	0.00	0.38	0.14	0.32	0.24
known.unknown.953.8	954	0.00	0.00	0.38	0.30	0.46	0.28	0.24
known.unknown.1630.0	1630	0.00	0.88	0.00	0.00	0.21	0.29	0.23
known.unknown.1278	1278	0.34	0.13	0.00	0.49	0.02	0.33	0.22
known.unknown.900.7	901	0.18	0.69	0.00	0.00	0.08	0.36	0.22
dodecanal	1410	0.40	0.05	0.00	0.21	0.10	0.46	0.20
1-tetradecene	1391	0.00	0.12	0.00	0.31	0.79	0.00	0.20
known.unknown.989.0	989	0.00	0.00	0.00	0.10	1.00	0.00	0.18
alloaromadendren *	1462	0.00	0.00	0.00	0.00	1.07	0.00	0.18
1-tridecene	1294	0.00	0.00	0.00	0.12	0.91	0.00	0.17
known.unknown.1747.6	1748	0.00	0.00	0.00	0.39	0.51	0.00	0.15
1-pentadecene	1491	0.07	0.00	0.00	0.13	0.66	0.00	0.14
α-phellanderen *	1003	0.00	0.83	0.00	0.00	0.00	0.00	0.14
hexadecane	1600	0.34	0.03	0.05	0.09	0.30	0.00	0.13
acetic acid, heptyl ester	1100	0.00	0.00	0.00	0.00	0.80	0.00	0.13
butylhexanoate *	1188	0.79	0.00	0.00	0.00	0.00	0.00	0.13
2-buten-1-ol, 3-methyl-, acetate	910	0.73	0.00	0.00	0.00	0.04	0.00	0.13
known.unknown.1271.5	1272	0.00	0.13	0.00	0.37	0.17	0.06	0.12
known.unknown.850.7	851	0.00	0.00	0.73	0.00	0.00	0.00	0.12
pseudocumene *	996	0.58	0.00	0.15	0.00	0.00	0.00	0.12
known.unknown.1497.0	1497	0.27	0.19	0.00	0.26	0.00	0.00	0.12
*cis*-3-hexenyl benzoate *	1568	0.00	0.48	0.00	0.00	0.24	0.00	0.12
(-)limonene *	1030	0.00	0.00	0.00	0.25	0.45	0.00	0.12
β-pinone	1138	0.12	0.40	0.00	0.00	0.11	0.00	0.11
known.unknown.1098.6	1099	0.15	0.32	0.00	0.00	0.15	0.00	0.10
known.unknown.1463.4	1463	0.00	0.00	0.00	0.30	0.32	0.00	0.10
known.unknown.1161	1161	0.33	0.15	0.12	0.00	0.00	0.00	0.10
known.unknown.1025.7	1026	0.24	0.04	0.32	0.00	0.00	0.00	0.10
known.unknown.1693.9	1694	0.00	0.20	0.00	0.00	0.39	0.00	0.10
known.unknown.1457.3	1458	0.00	0.00	0.00	0.25	0.35	0.00	0.10
known.unknown.1547.9	1548	0.00	0.00	0.00	0.59	0.00	0.00	0.10
known.unknown.1458.6	1459	0.00	0.57	0.00	0.00	0.00	0.00	0.10
2-decenal, (Z)-	1266	0.02	0.30	0.00	0.00	0.25	0.00	0.10
*cis*-3-hexenyl acetate II	1011	0.16	0.04	0.03	0.00	0.34	0.00	0.09
known.unknown.1067	1067	0.15	0.06	0.00	0.18	0.02	0.14	0.09
1-dodecene	1192	0.00	0.00	0.00	0.00	0.55	0.00	0.09
known.unknown.1092.0	1092	0.00	0.00	0.00	0.00	0.51	0.00	0.09
known.unknown.1474.6	1475	0.00	0.42	0.00	0.00	0.08	0.00	0.08
β-Myrcene	992	0.00	0.09	0.00	0.41	0.00	0.00	0.08
dodecane	1200	0.13	0.11	0.03	0.00	0.23	0.00	0.08
octanoic acid	1179	0.08	0.17	0.00	0.00	0.23	0.00	0.08
hexanoic.acid	988	0.00	0.41	0.00	0.00	0.05	0.00	0.08
germacrene D *	1480	0.00	0.00	0.30	0.00	0.16	0.00	0.08
β-elemene	1394	0.00	0.00	0.19	0.00	0.26	0.00	0.07
known.unknown.1433	1433	0.00	0.00	0.00	0.00	0.42	0.00	0.07
known.unknown.1094	1094	0.20	0.21	0.00	0.00	0.00	0.00	0.07
known.unknown.1705	1705	0.00	0.16	0.06	0.00	0.18	0.00	0.07
known.unkown.1298.0	1298	0.00	0.00	0.38	0.00	0.00	0.00	0.06
known.unknown.1031.7	1032	0.00	0.05	0.00	0.30	0.04	0.00	0.06
known.unknown.1496.2	1492	0.00	0.21	0.00	0.00	0.16	0.00	0.06
known.unknown.1094.9	1095	0.34	0.00	0.00	0.00	0.02	0.00	0.06
1-nonene	892	0.00	0.00	0.00	0.00	0.35	0.00	0.06
known.unknown.1460.7	1461	0.00	0.14	0.00	0.00	0.07	0.13	0.06
known.unknown.1025.2	1025	0.00	0.32	0.00	0.00	0.00	0.00	0.05
known.unknown.988.8	989	0.00	0.20	0.00	0.00	0.00	0.12	0.05
*trans*-2-octenal *	1056	0.00	0.25	0.00	0.00	0.06	0.00	0.05
known.unknown.1692.9	1693	0.03	0.00	0.00	0.00	0.28	0.00	0.05
known.unknown.1340.7	1341	0.00	0.16	0.00	0.00	0.14	0.00	0.05
known.unknown.1050.0	1050	0.00	0.07	0.06	0.00	0.16	0.00	0.05
3-octadecene, (*E*)-	1795	0.00	0.00	0.00	0.00	0.29	0.00	0.05
octadecane	1800	0.10	0.05	0.03	0.00	0.06	0.00	0.04
nonadecane	1900	0.09	0.03	0.03	0.00	0.09	0.00	0.04
*trans*-2-hexenyl acetate *	1005	0.00	0.19	0.05	0.00	0.00	0.00	0.04
2-heptanone *	890	0.00	0.03	0.00	0.00	0.20	0.00	0.04
known.unknown.1381.3	1381	0.00	0.07	0.00	0.00	0.16	0.00	0.04
known.unknown.1527.0	1527	0.00	0.00	0.00	0.00	0.21	0.00	0.03
benzeneacetaldehyde	1043	0.00	0.17	0.00	0.00	0.03	0.00	0.03
known.unknown.1599.8	1600	0.00	0.09	0.00	0.00	0.08	0.00	0.03
2-decanone	1190	0.00	0.00	0.00	0.00	0.17	0.00	0.03
known.unknown.1025.8	1026	0.00	0.08	0.00	0.00	0.08	0.00	0.03
terpinolene *	1087	0.00	0.00	0.00	0.00	0.14	0.00	0.02
known.unknown.1163.9	1164	0.00	0.14	0.00	0.00	0.00	0.00	0.02
*cis*-3-hexenyl isovalerate	1235	0.00	0.12	0.00	0.00	0.00	0.00	0.02
γ-terpinene *	1050	0.00	0.00	0.00	0.00	0.10	0.00	0.02
known.unknown.1036.8	1037	0.00	0.06	0.00	0.00	0.03	0.00	0.02

Bold-printed compounds were used for EAG and/or olfactometer tests; * VOCs identified based on the comparison of retention index and mass spectra data with an authentic standard.
